# Supplementary material for: Diminished Signal‐to‐Noise Ratio Disrupts Somatosensory Population Encoding and Drives Tactile Hyposensitivity in the Fmr1−/y Autism Model
Source: Adv Sci (Weinh). 2026 Apr 15;13(28):e19479. doi: 10.1002/advs.202519479 (PMC13185836; doi:10.1002/advs.202519479)
Supplement: Supplementary file 1 — Supporting File: advs73741‐sup‐0001‐SuppMat.docx. [file ADVS-13-e19479-s001.docx]

**Supplemental data**

**
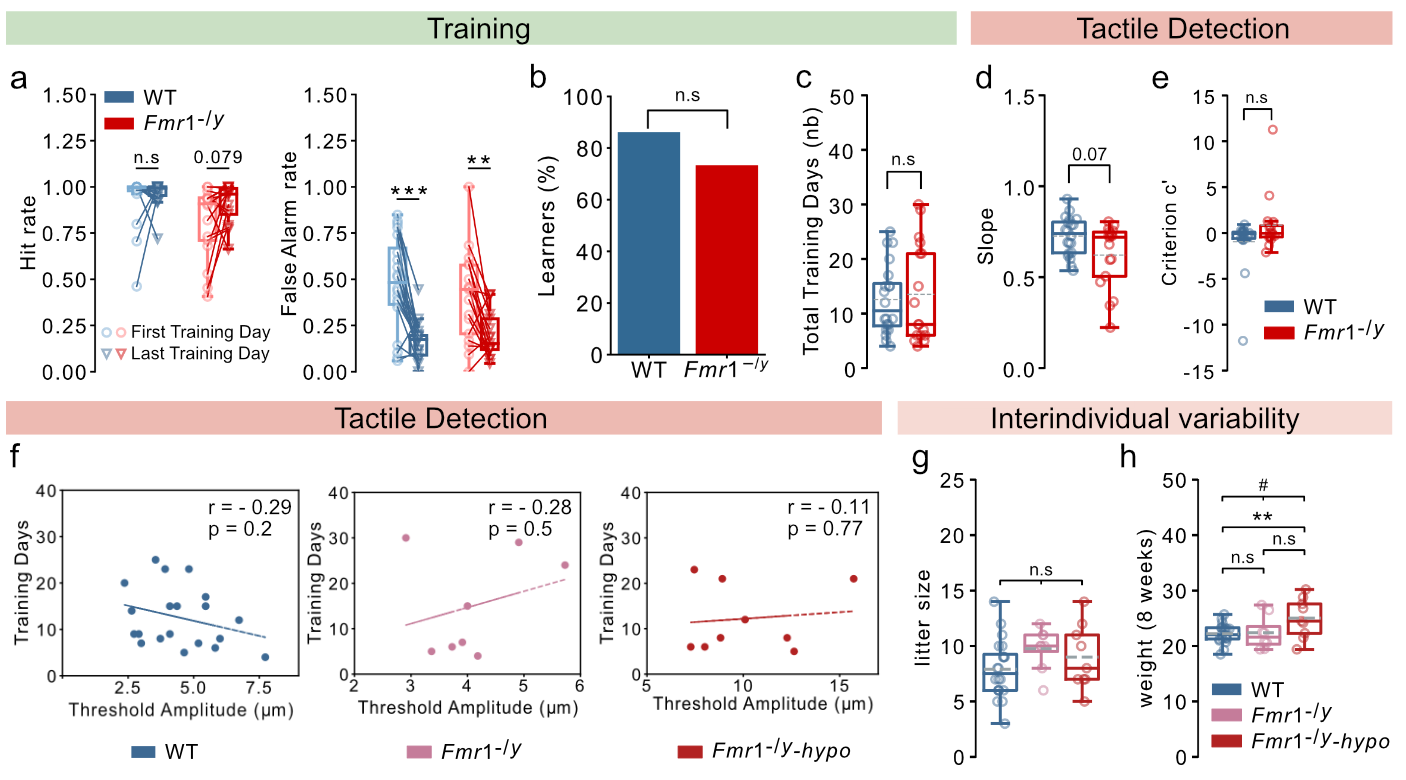
**

**Figure S1.** **Learning, tactile detection, and subgroup features**

For panels **a, c, d, f, g, h,**: n=20 WT, 17 *Fmr1*^-/y^ mice (of which 9 *Fmr1*^-/y^-hyposensitive). For panel **b**,: n=29 WT, 30 *Fmr1*^-/y^ mice. For panel **e,**: n=19 WT, 16 *Fmr1*^-/y^ mice. **a,** Hit rate (left) and False Alarm rate (right) during the first day of training (following pre-training, see Methods; light-color, circles) and the last day of training (dark color, triangles) for all mice that underwent detection testing. **b,** Percentage of mice that reached the learning criterion. **c,** Total number of days spent in pre-training and training for all the animals that underwent detection testing. **d,** Perceptual accuracy calculated as the slope of the psychometric curve for each mouse. **e,** Licking strategy calculated as relative criterion c’ for each mouse. **f,** Correlation of the total number of pre-training and training days with the threshold amplitude for WT (left), typically-detecting *Fmr1*^-/y^ mice (middle), and *Fmr1*^-/y^-hyposensitive mice (right). **g,** Size of the litter each mouse was born in. **h,** Weight of each mouse at 8 weeks before water restriction. P values were computed using a Wilcoxon signed-rank test and two-sided paired t-test for panel **a,;** a Chi square for panel **b,**; Mann-Whitney U test for panels **c,** **d,** **e,;** Pearson correlation coefficient for panel **f,;** and an one-way ANOVA and two-sided t-test for panels **g, h**,***P < 0.001, **P < 0.01 ^#^P < 0.05, or n.s, not significant. ^#^ indicates ANOVA results.


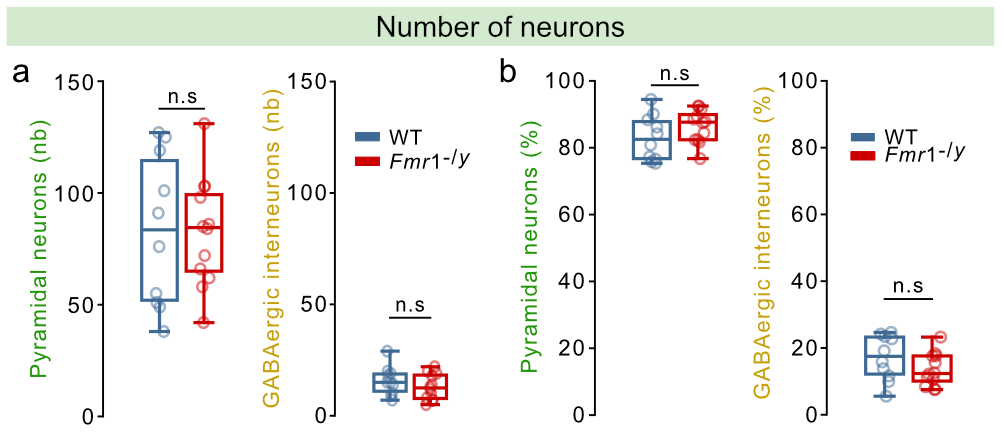


**Figure S2.** **Number of recorded neurons.** For all panels n=10 WT, 12 *Fmr1*^-/y^ mice. **a,** Number of pyramidal neurons (left) and GABAergic interneurons (right) detected in the field of view as regions of interest (ROIs) after manual curation. **b,** Same as **a,** but expressed as percentage of the transfected neurons in the field of view. P values were computed using two-sided t-test for all panels. n.s, not significant.


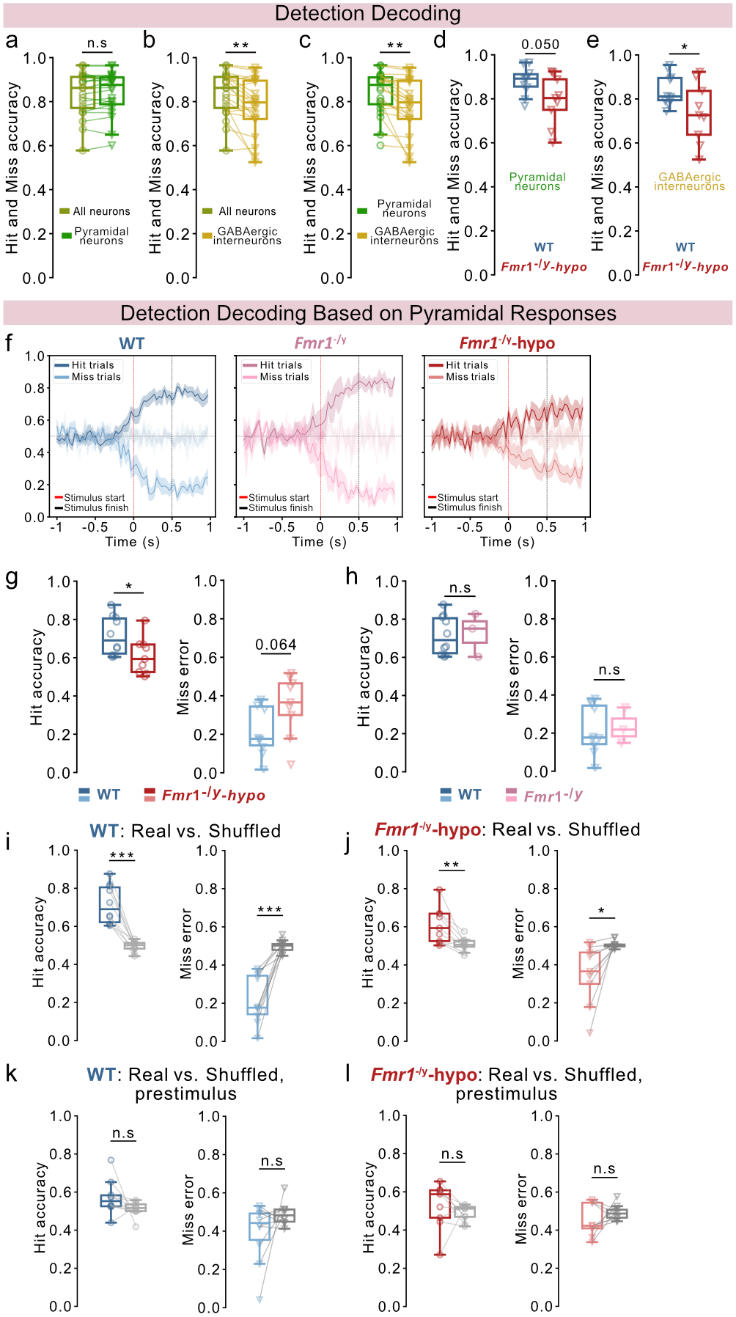


**Figure S3. Detection decoding in S1-FP based on pyramidal neurons or GABAergic interneurons.** For panels **a-c**: 19 mice (10 WT, 9 *Fmr1*^-/y^–hyposensitive mice). For panels **d, e,**: n=10 WT, 9 *Fmr1*^-/y^–hyposensitive mice. ~10 repetitions of each amplitude per mouse. Classifiers were trained on the mean neuronal activity during stimulation (500 ms) to decode if the trial was detected (Hit) or non-detected (Miss). **a,** Comparison of decoding accuracy for Hit and Miss trials when training the model with all neurons versus only pyramidal neurons. **b**, Same as **a,** but comparing the model’s accuracy when trained with all neurons versus only with GABAergic interneurons. **c**, Same as **a,** but comparing the model’s accuracy when trained with pyramidal neurons versus with GABAergic interneurons. **d**, Decoding accuracy for Hit and Miss trials when training the model with pyramidal neurons in WT and *Fmr1*^-/y^-hyposensitive mice. **e**, Same as **d**, but using only GABAergic interneurons. **f,** Response decoding in each mouse group based on pyramidal responses. Stimulus start is indicated by a red dashed line and stimulus end by a black dashed line. The dark line represents true Hit classification and the light line represents false Miss (Miss error) classification. The very light traces near y = 0.5 represent the model accuracy for hit and miss when trained on shuffled-label datasets and provide the chance-level baseline. Datasets including prestimulus activity (1 s), tactile stimulation (500 ms), post-stimulus activity (500 ms) of pyramidal neurons were used to train the logistic regression model. Classifiers were trained on each frame to decode if the trial was detected (Hit) or non-detected (Miss) (adapted from ref^44^). Comparison of the average Hit accuracy (left) and Miss error (right) of the classifiers during stimulation between WT and *Fmr1*^-/y^-hyposensitive mice **(g,)** and between WT and *Fmr1*^-/y^ mice with intact detection thresholds **(h,).** Average Hit accuracy (left) and Miss error (right) of the classifiers on the total duration of the stimulation (500 ms) compared to the accuracy of the model trained with shuffled behavioral labels in WT mice **(i,)** and *Fmr1*^-/y^-hyposensitive mice **(j,)**. Average Hit accuracy (left) and Miss error (right) of the classifiers on the prestimulus activity (200 ms) compared to the accuracy of the model trained with shuffled behavioral labels in WT mice **(k,)** and *Fmr1*^-/y^-hyposensitive mice **(l,).** P values were computed using two-sided paired t-test for panels **a, b, c, i, j, k-left, l**; Wilcoxon signed-rank test for the panel **k-right,** and two-sided t-test for panels **d, e, g, h,**. ***P < 0.001, **P < 0.01, *P < 0.05, or n.s, not significant.


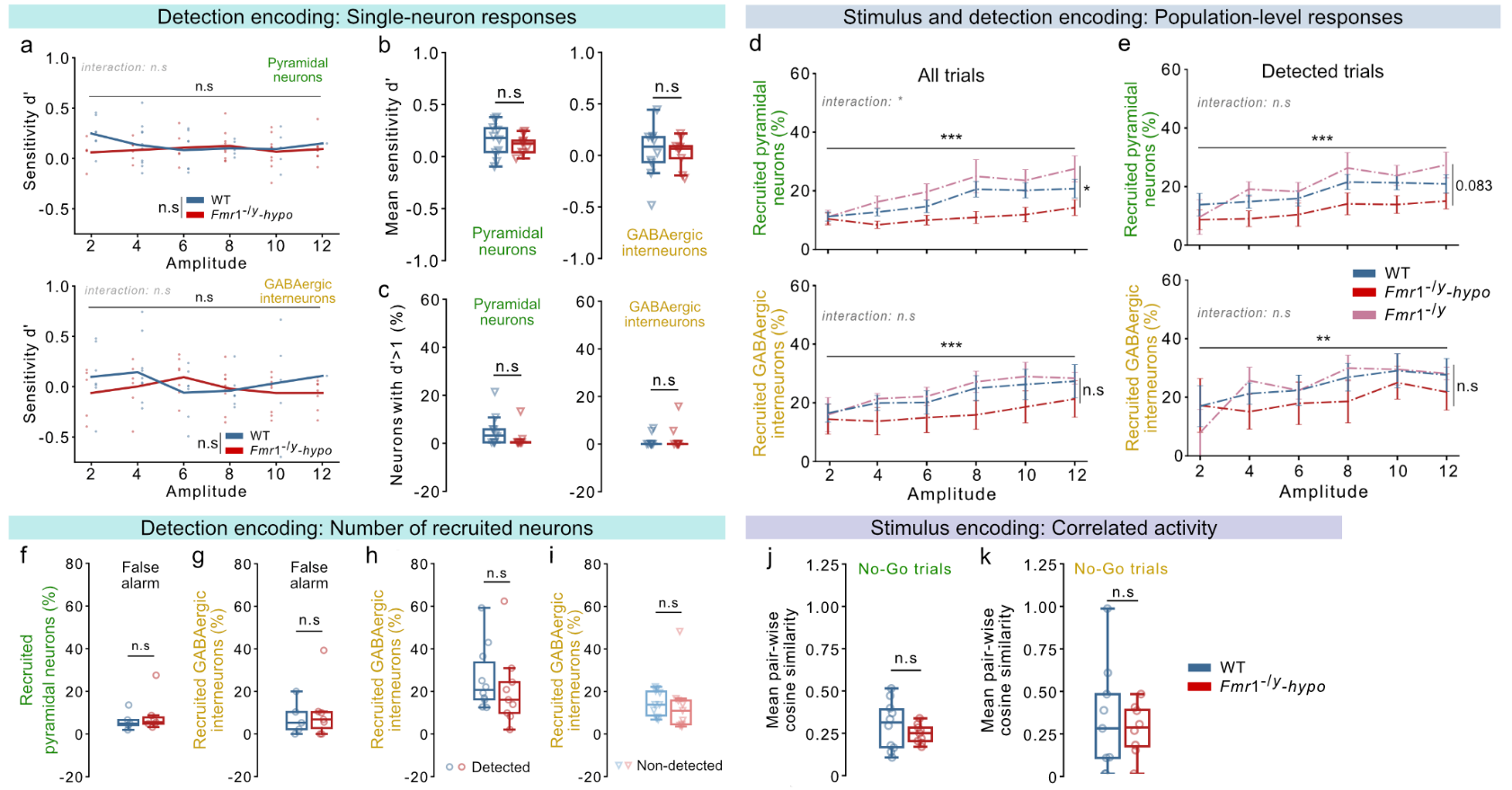


**Figure S4. Stimulus and detection encoding in S1-FP.**

For panels **a**: n=1-10 WT, 5-9 *Fmr1*^-/y^-hyposensitive mice, depending on how many mice had detected and non-detected trials at each stimulus amplitude. For panels **b, c, h, i, j**: n=10 WT, 9 *Fmr1*^-/y^-hyposensitive mice. For panels **d-e**: n=10 WT, 3 *Fmr1*^-/y^ mice with intact detection thresholds, 9 *Fmr1*^-/y^-hyposensitive mice. For panels **f, g,**: n=5 WT, 7 *Fmr1*^-/y^-hyposensitive mice. For panel **k,**: n=9 WT, 8 *Fmr1*^-/y^-hyposensitive mice. 1 session of ~10 repetitions of each amplitude per mouse. **a,** Mean single-neuron detection sensitivity (d’) for stimulus-recruited pyramidal neurons (top) and GABAergic interneurons (bottom) at each stimulus amplitude. **b,** Same as **a,** but with all stimulus amplitudes grouped together. **c,** Proportion of pyramidal neurons (left) and GABAergic interneurons (right) with detection sensitivity d’>1. **d,** Proportion of pyramidal neurons (top) and GABAergic interneurons (bottom) recruited (activated or inhibited) during stimulus delivery at each different amplitude. **e,** Same as **d,** but during stimulus delivery only for detected trials of each amplitude. **f,** Proportion of recruited pyramidal neurons during False Alarm No-Go trials. **g,** Same as **f,** but for GABAergic interneurons. **h,** Proportion of recruited GABAergic interneurons during detected stimuli. **i,** Same as **h,** but for non-detected stimuli. **j,** Mean pair-wise cosine similarity of the trial-by-trial responses of pyramidal neurons during No-Go (catch) trials. **k,** Mean pair-wise cosine similarity of the trial-by-trial responses of GABAergic interneurons during No-Go (catch) trials. P values were computed using a Mixed ANOVA for panel **a,**; a Mixed ANOVA after a Yeo-Johnson transformation of the data for panels **d, e,**; two-sided t-test for panels **b, h, j, k,**; Mann-Whitney U test for panels **c, f, g, i,**. ***P < 0.001, **P < 0.01, or n.s, not significant.


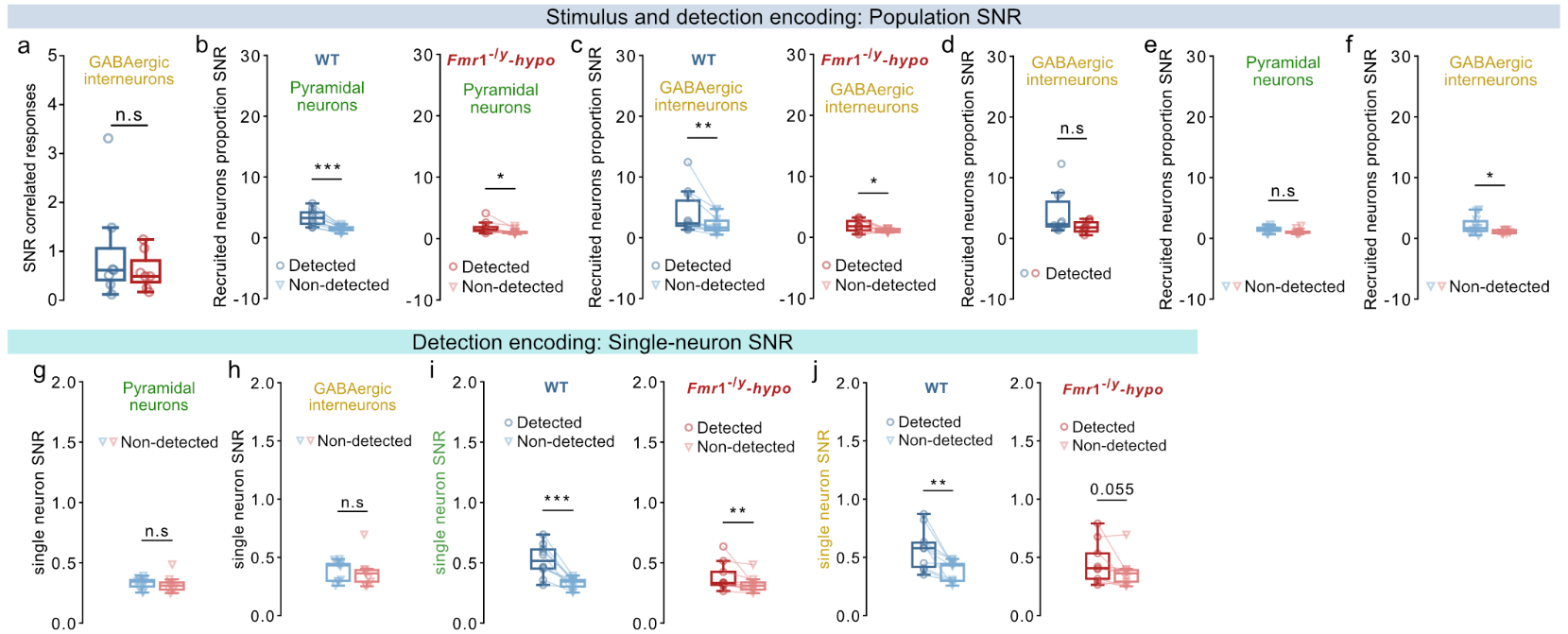


**Figure S5. Signal-to-noise ratio (SNR) during stimulus and detection encoding.**

For panel **a,**: n=7 WT, 7 *Fmr1*^-/y^-hyposensitive mice. For all other panels: n=10 WT, 9 *Fmr1*^-/y^-hyposensitive mice; 1 session of ~10 repetitions of each amplitude per mouse. **a,** Ratio of the mean pair-wise correlation of GABAergic interneurons during the tactile stimulus and during No-Go (catch) trials. **b,** Comparison of the ratio of recruited pyramidal neurons during detected trials to No-Go (catch) trials versus the ratio during non-detected trials to No-Go trials in WT (left) and *Fmr1*^-/y^-hyposensitive mice (right). **c,** Same as **b,** but for recruited GABAergic interneurons. **d,** Ratio of the proportion of recruited GABAergic interneurons during detected trials and during No-Go (catch) trials. **e,** Ratio of the proportion of recruited pyramidal neurons during non-detected trials and during No-Go (catch) trials. **f,** Same as **e,** but for GABAergic interneurons. **g,** Difference between the average z-score response of pyramidal neurons during non-detected trials and during No-Go (catch) trials. **h,** Same as **g,** but for GABAergic interneurons. **i,** Comparison of the difference between the average z-score response of pyramidal neurons during detected trials to No-Go (catch) trials versus the difference during non-detected trials to No-Go trials in WT (left) and *Fmr1*^-/y^-hyposensitive mice (right). **j,** Same as **i,** but for GABAergic interneurons. P values were computed using two-sided t-test for panels **f, g,**; a Wilcoxon signed-rank test for panels **b-right, c-left, i-right, j-right,**; two-sided paired t-test for panels **b-left, c-right, i-left, j-left ,**; Mann-Whitney U test for panels **a, d, e, h,**. ***P < 0.001, **P < 0.01, *P < 0.05, or n.s, not significant.
